# Baseline ASPECTS, Fibrinogen Level, and Platelet Count for Predicting Hemorrhagic Transformation After Mechanical Thrombectomy in Acute Ischemic Stroke

**DOI:** 10.3390/brainsci16070704

**Published:** 2026-06-30

**Authors:** Nguyen Van Tuyen, Nguyen Van Tuan, Tran Tien Dung, Nguyen Ngoc Hien, Le Chi Vien, Nguyen Cam Thach, Nguyen Hoang Ngoc, Le Duy Cuong

**Affiliations:** 1 Department of Stroke, 108 Military Central Hospital, 1 Tran Hung Dao Street, Hai Ba Trung Ward, Hanoi 10000, Vietnam; bstuyena21@gmail.com (N.V.T.); nguyenvantuan98lcs@gmail.com (N.V.T.); tiendungg77@gmail.com (T.T.D.); hienhien.hvqy@gmail.com (N.N.H.); dr.chivien.bv108@gmail.com (L.C.V.); 2 VNU University of Medicine and Pharmacy, Hanoi 10000, Vietnam; 3 Department of Biochemistry, Medical Laboratory Center, 108 Military Central Hospital, 1 Tran Hung Dao Street, Hai Ba Trung Ward, Hanoi 10000, Vietnam; nguyencamthach1973@yahoo.com; 4 Department of Neurology, 108 Military Central Hospital, 1 Tran Hung Dao Street, Hai Ba Trung Ward, Hanoi 10000, Vietnam; hoangngocdqn108@gmail.com; 5 Department of Experimental Medicine, 108 Military Central Hospital, 1 Tran Hung Dao Street, Hai Ba Trung Ward, Hanoi 10000, Vietnam

**Keywords:** hemorrhagic transformation, acute ischemic stroke, fibrinogen, platelet

## Abstract

**Highlights:**

**What are the main findings?**

- Baseline ASPECTS, fibrinogen level, and platelet count were independently associated with hemorrhagic transformation after mechanical thrombectomy in patients with acute ischemic stroke.
- A reduced predictive model incorporating baseline ASPECTS, fibrinogen level, and platelet count demonstrated acceptable discriminative performance comparable to the full model while maintaining model parsimony.

**What are the implications of the main findings?**

- Combining neuroimaging and hematologic biomarkers may improve early risk stratification for hemorrhagic transformation after mechanical thrombectomy.
- The reduced model may provide a simple and clinically applicable approach for identifying patients at increased risk of post-thrombectomy hemorrhagic transformation, although external validation is required.

**Abstract:**

***Background:*** Hemorrhagic transformation (HT) is a major complication after mechanical thrombectomy (MT) in acute ischemic stroke (AIS). This study evaluated the predictive value of baseline ASPECTS, fibrinogen level, and platelet count for HT after MT. ***Methods:*** We retrospectively analyzed 274 AIS patients treated with MT. HT within 24 h was identified using follow-up imaging. Logistic regression, ROC analysis, DeLong tests, and bootstrap resampling (1000 iterations) were performed to evaluate predictors and model performance. ***Results:*** HT occurred in 52/274 patients (19.0%). Baseline ASPECTS (adjusted OR 0.73, 95% CI 0.61–0.88; *p* = 0.001), fibrinogen level (adjusted OR 0.51, 95% CI 0.32–0.79; *p* = 0.003), and platelet count (adjusted OR 0.99, 95% CI 0.99–1.00; *p* = 0.018) were independently associated with HT. Baseline ASPECTS showed the highest discriminative ability among individual predictors (AUC = 0.684), whereas baseline NIHSS score showed poor performance (AUC = 0.539). The reduced model combining baseline ASPECTS score, fibrinogen level, and platelet count achieved a balanced discriminative performance (AUC = 0.739), with no significant difference compared with the full model (*p* = 0.804) or baseline ASPECTS alone (*p* = 0.112). Similarly, the full model did not significantly outperform baseline ASPECTS (*p* = 0.120). Bootstrap validation confirmed model stability. ***Conclusions:*** Baseline ASPECTS, fibrinogen level, and platelet count are independent predictors of HT after MT. A reduced model incorporating these variables demonstrated modest-to-acceptable and parsimonious predictive performance, although external validation is required before clinical implementation.

## 1. Introduction

Acute ischemic stroke (AIS) remains a leading cause of long-term disability in developed countries and represents a major public health burden worldwide. It imposes substantial clinical, economic, and social consequences on patients, families, and healthcare systems [1]. According to the Global Burden of Disease 2021 report, the global burden of ischemic stroke remains considerable, with nearly 70 million prevalent cases and an incidence rate of 819.5 per 100,000 population [2]. The incidence and prevalence of AIS continue to increase, in the context of heterogeneous and unevenly distributed risk factors across populations [3]. Since 2015, robust evidence from pivotal randomized controlled trials has established mechanical thrombectomy (MT) as a highly effective reperfusion strategy for patients with large vessel occlusion. Since then, significant advances have been made in defining and expanding treatment eligibility criteria. The superiority of MT combined with standard medical therapy over medical therapy alone in selected patients with large vessel occlusion has been consistently demonstrated [4,5]. However, reperfusion therapies—whether pharmacological thrombolysis or mechanical thrombectomy—are not without limitations. Strict time constraints, multiple contraindications, and procedure-related complications restrict their universal applicability. Among these complications, hemorrhagic transformation (HT) represents one of the most severe and clinically consequential adverse events following cerebral reperfusion [6]. Reported rates of HT vary widely across studies, with experimental and clinical data suggesting incidences exceeding 50% in certain high-risk populations [7,8]. Therefore, identifying prognostic factors associated with HT has become an important research focus to improve patient selection, optimize procedural safety, and enhance clinical outcomes after MT. Among imaging-based predictors, the Alberta Stroke Program Early CT Score (ASPECTS) has been widely used to estimate infarct burden and predict clinical outcomes after reperfusion therapy [9,10]. Lower ASPECTS values have been associated with an increased risk of HT and poor functional outcomes following MT [11].

Numerous studies have investigated the risk factors associated with HT following MT in patients with AIS [6,12,13,14,15]. Recently, increasing attention has been directed toward prognostic biomarkers for HT, particularly platelet count and plasma fibrinogen level. The role of platelet count as a predictor of HT following thrombolytic therapy has been investigated, yielding inconsistent findings. Some studies suggest that lower platelet counts are not significantly associated with an increased risk of HT [16,17]. Conversely, another study reported that lower platelet counts were associated with an increased risk of HT following thrombolytic therapy [18]. Moreover, the 2018 clinical guidelines issued by the American Heart Association/American Stroke Association do not recommend reperfusion therapy in patients with a platelet count < 100,000/mm^3^ [19]. In addition, the fibrinogen levels have been investigated and shown to be closely associated with stroke outcomes, including the risk of hemorrhagic transformation, with variable sensitivity and specificity across studies. This study reported that a fibrinogen level < 1.50 g/L was an independent risk factor for hemorrhagic transformation following thrombolytic therapy. The fibrinogen level has been demonstrated to be closely associated with the risk of hemorrhagic transformation, with varying degrees of specificity across studies. Wang et al. (2019) found that a fibrinogen level < 1.50 g/L was a risk factor for hemorrhagic transformation following thrombolytic therapy [15]. Vandelli et al. (2015) reported that a post-thrombolysis reduction in the fibrinogen level < 2 g/L, or a decrease of ≥25%, was associated with an increased risk of intracranial hemorrhage [20]. Yan et al. (2019) found that an early decrease in the fibrinogen level was associated with sICH following reperfusion therapy with intravenous thrombolysis, with or without endovascular thrombectomy [21]. However, another study demonstrated that a change in fibrinogen levels > 2 g/L was an independent predictor of symptomatic intracranial hemorrhage (sICH) [22].

Moreover, studies investigating the combined prognostic value of the platelet count and fibrinogen level in predicting HT after MT in patients with AIS remain relatively limited. This gap highlights the need for further research to clarify the predictive utility of these hematological biomarkers for HT in patients with AIS undergoing MT. Therefore, this study aimed to evaluate the combined predictive value of fibrinogen level and platelet count for HT after MT in patients with AIS and to assess their incremental value beyond imaging-based predictors.

## 2. Materials and Methods

### 2.1. Study Population

This study enrolled patients with AIS due to large artery occlusion in the anterior circulation who underwent MT and achieved vascular recanalization at the Department of Neurointervention, 108 Military Central Hospital, Hanoi, Vietnam, between November 2023 and September 2025.

Inclusion criteria were as follows: (1) provision of informed consent to participate in the study; (2) patients with occlusion of a large artery in the anterior circulation confirmed by computed tomography angiography (CTA); (3) MT performed within 24 h of symptom onset, pateints with or without intravenous thrombolysis, ASPECTS ≥ 6, NIHSS ≥ 5; (4) availability of pre-intervention blood test results; and (5) follow-up CT imaging within 24 h after MT for evaluation of HT.

Exclusion criteria: (1) patients with incomplete clinical data; (2) missing laboratory results, or absence of follow—up CT imaging; (3) patients with severe comorbid organ dysfunction, including severe heart failure (New York Heart Association [NYHA] class III–IV), decompensated hepatic disease, or severe renal insufficiency (estimated glomerular filtration rate [eGFR] < 30 mL/min/1.73 m^2^ or requiring dialysis).

### 2.2. Methods

#### 2.2.1. Study Design

This retrospective observational cohort study included 274 consecutive patients who met the predefined inclusion and exclusion criteria during the study period.

#### 2.2.2. Blood Sampling and Laboratory Testing

Baseline laboratory parameters, including hematological parameters (complete blood count), coagulation profile, fibrinogen level, admission glucose level, serum electrolytes, and liver and renal function tests, were obtained immediately upon hospital admission before mechanical thrombectomy. Other metabolic parameters requiring fasting conditions were assessed separately when applicable. Biochemical parameters were measured using the Architect c16000 system (Abbott, Tokyo, Japan) and three Architect i2000SR chemiluminescent immunoassay analyzers (Abbott, Abbott Park, IL, USA). Hematological and coagulation parameters were measured using the ACL TOP 700 system (Instrumentation Laboratory, Werfen, Bedford, MA, USA).

#### 2.2.3. Endovascular Therapy

Participants were treated at the Department of Neurointervention, a comprehensive stroke center with 24/7 endovascular capability. Treatment decisions were made by a multidisciplinary stroke team comprising neurologists, neurointerventionalists, and neuroradiologists. MT was performed according to two strategies: (1) bridging therapy in patients who had received intravenous alteplase prior to endovascular intervention, or (2) direct MT in patients with contraindications to intravenous thrombolysis or those presenting beyond the therapeutic time window.

The endovascular procedure was performed via the transfemoral arterial approach using a standard guiding catheter. Thrombectomy devices included a stent retriever, a large-bore aspiration catheter, or a combination of both techniques, depending on lesion characteristics and the operator’s experience. In cases of tandem lesions, such as extracranial internal carotid artery stenosis or dissection, individualized interventional strategies (including balloon angioplasty and/or stent placement) were considered to ensure optimal cerebral reperfusion. The degree of reperfusion was assessed using the modified Thrombolysis in Cerebral Infarction (mTICI) scale, with successful recanalization defined as an mTICI score ≥ 2b.

#### 2.2.4. Follow-Up and Outcome Assessment

Follow-up CT was performed within 24 h after the procedure or earlier if patients exhibited clinical deterioration. Imaging assessments were conducted independently by two experienced neurologists or neuroradiologists who were not directly involved in the intervention and were blinded to the patients’ laboratory results. In cases of disagreement, a final consensus was reached through discussion or consultation with a third senior expert.

HT was classified according to the European Cooperative Acute Stroke Study II (ECASS II) criteria [23], including hemorrhagic infarction (HI1 and HI2) and parenchymal hematoma (PH1 and PH2). Symptomatic intracranial hemorrhage (sICH) was defined as intracranial bleeding accompanied by clinical deterioration, indicated by an increase of ≥4 points in the National Institutes of Health Stroke Scale (NIHSS) score compared with baseline, and not attributable to other causes.

The primary outcome of the study was the occurrence of HT following MT. Secondary outcomes included sICH, in-hospital mortality, and functional outcome at 90 days after stroke onset, assessed using the modified Rankin Scale (mRS). A favorable functional outcome was defined as an mRS score of 0–2 [24,25].

#### 2.2.5. Statistical Analysis

All statistical analyses were performed using SPSS version 26.0 (IBM Corp., Armonk, NY, USA) and MedCalc version 23.5.3 (MedCalc Software Ltd., Ostend, Belgium). A two-sided *p*-value < 0.05 was considered statistically significant. Continuous variables were assessed for normality using the Shapiro–Wilk test and were expressed as median (interquartile range, IQR). Comparisons between groups (hemorrhagic transformation vs. non-hemorrhagic transformation) were performed using the Mann–Whitney U test. Categorical variables were presented as frequencies and percentages and compared using the chi-square test or Fisher’s exact test. To account for multiple comparisons in baseline characteristics, *p*-values were adjusted using the Benjamini–Hochberg false discovery rate procedure.

Variables with *p* < 0.10 in univariate analysis, together with clinically relevant variables, were entered into a multivariable logistic regression model to identify independent predictors of hemorrhagic transformation [26,27]. A backward stepwise selection method was applied to obtain the final model. Results were reported as odds ratios (ORs) with 95% confidence intervals (CIs). Receiver operating characteristic (ROC) curve analysis was conducted using MedCalc to evaluate the discriminative ability of individual predictors (fibrinogen level, platelet count, baseline NIHSS score, and baseline ASPECTS) as well as the final combined model. The area under the curve (AUC) with 95%CI was calculated. Comparisons between ROC curves were performed using the DeLong test [28].

## 3. Results

### 3.1. Baseline Characteristics of Participants

In Table 1, 52/274 (19.0%) patients with AIS developed HT within the first 24 h after MT. Patients with HT had significantly lower fibrinogen level (*p* = 0.001), significantly lower platelet count (*p* = 0.002), a higher history of dyslipidemia (*p* = 0.038), and lower blood glucose level (*p* = 0.033) compared with those without HT. In addition, the HT group demonstrated significantly higher NIHSS scores at day 10 after MT (*p* < 0.001), lower ASPECTS on admission (*p* < 0.001), and higher modified Rankin Scale (mRS) score at discharge (*p* = 0.001) compared with those without HT. No statistically significant differences were observed between the two groups with respect to other baseline characteristics (*p* > 0.05). However, after Benjamini–Hochberg adjustment for multiple comparisons, only baseline ASPECTS, 10-day NIHSS score, discharge mRS score, platelet count, and fibrinogen level remained significantly different between the two groups (all adjusted *p* < 0.05).

HI in 21/52 patients (40.4%) and PH in 31/52 patients (59.6%), sICH occurred in 14/52 patients with HT (26.9%), and in-hospital mortality occurred in 9/52 patients with HT (17.3%). The study compared baseline characteristics between the two HT subgroups: A statistically significant difference was observed between the two HT subgroups in NIHSS score at day 10 after MT (*p* = 0.007) and mRS score at discharge (*p* = 0.017) (Table 2). Following Benjamini–Hochberg correction for multiple comparisons, no variables remained statistically significant (all adjusted *p*-values > 0.05).

### 3.2. Risk Factors for HT in AIS Patients After MT

Variables with *p* < 0.10 in the univariate analysis or with clinical relevance were considered for multivariable logistic regression. To minimize overfitting given the limited number of events, a parsimonious model was constructed. In the multivariable analysis, baseline ASPECTS (OR 0.73, 95% CI 0.61–0.88; *p* = 0.001), fibrinogen level (OR 0.51, 95% CI 0.32–0.79; *p* = 0.003) and platelet count (OR 0.99, 95% CI 0.99–1.0; *p* = 0.018) were independently associated with HT. Higher ASPECTS, fibrinogen level, and platelet count were associated with lower odds of HT. The corresponding reduced prediction model was defined as logit(PHT) = 4.920 − 0.310 × baseline ASPECTS − 0.007 × platelet count (×10^9^/L) − 0.683 × fibrinogen level (g/L) (Table 3). The calibration of the reduced logistic regression model was assessed using the Hosmer–Lemeshow goodness-of-fit test, which indicated good model fit (χ^2^ = 9.426, df = 8, *p* = 0.308), suggesting no significant difference between observed and predicted probabilities.

Internal validation using bootstrap resampling (1000 iterations) demonstrated the stability of the multivariable logistic regression model, with consistent effect estimates across resamples. The bootstrap 95% confidence intervals for all predictors did not include zero, indicating robust associations.

In the bootstrap analysis, fibrinogen level (B = −0.683, 95% CI −1.119 to −0.294; *p* = 0.002), platelet count (B = −0.007, 95% CI −0.012 to −0.002; *p* = 0.016), and baseline ASPECTS (B = −0.310, 95% CI −0.505 to −0.167; *p* = 0.001) remained significantly associated with HT. Lower values of these variables were associated with higher odds of HT, supporting the robustness of the multivariable model (Table 4).

Internal validation using bootstrap resampling (1000 iterations) showed that the regression coefficients were stable, with small bias and consistent confidence intervals. The direction and magnitude of the associations for baseline ASPECTS, fibrinogen level, and platelet count were preserved, supporting the internal validity of the final model (Table 5).

ROC analysis was performed to evaluate the discriminative ability of individual markers for predicting HT after mechanical thrombectomy. Baseline NIHSS score was additionally included due to its established clinical relevance in AIS. Baseline ASPECTS demonstrated the highest discriminative ability (AUC = 0.684; 95% CI 0.625–0.738), followed by fibrinogen level (AUC = 0.650; 95% CI 0.590–0.706) and platelet count (AUC = 0.639; 95% CI 0.579–0.696), while baseline NIHSS showed low discriminative ability (AUC = 0.539; 95% CI 0.478–0.599) (Table 6).

**Figure 1:**
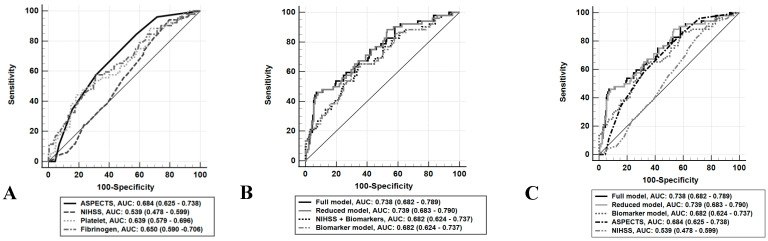
ROC curves for prediction of HT after MT in patients with AIS. (**A**) ROC curves of individual predictors, including baseline ASPECTS, baseline NIHSS score, fibrinogen level, and platelet count. (**B**) ROC curves of multivariable models, including the full model (baseline ASPECTS + baseline NIHSS score + fibrinogen level + platelet count), reduced model (baseline ASPECTS + fibrinogen level + platelet count), NIHSS + biomarker model (baseline NIHSS score + fibrinogen level + platelet count), and biomarker model (fibrinogen level + platelet count). (**C**) ROC curves of multivariable models, including the reduced model, full model, baseline ASPECTS, biomarker model, and baseline NIHSS score.

As shown in Figure 1A, among individual predictors, baseline ASPECTS exhibited the highest discriminative ability, whereas baseline NIHSS score showed the lowest performance. Fibrinogen and platelet count demonstrated comparable and moderate discrimination.

In Figure 1B, all multivariable models showed improved performance compared with individual predictors. The full and reduced models achieved the highest AUC values, with no meaningful difference between them, supporting the parsimonious nature of the reduced model.

Figure 1C provides an overall comparison of all models, confirming that the full and reduced models had comparable and superior discriminative performance relative to individual predictors. Baseline ASPECTS and the biomarker model showed moderate and similar performance, while NIHSS demonstrated the lowest discriminative ability.

Pairwise comparisons using DeLong’s test demonstrated that Baseline ASPECTS had significantly higher discriminative ability than the baseline NIHSS score (ΔAUC = 0.145, *p* = 0.002). In contrast, no statistically significant differences were observed between Baseline ASPECTS and fibrinogen level (*p* = 0.519) or platelet count (*p* = 0.407), indicating comparable predictive performance among these markers. Fibrinogen level showed significantly better performance than the baseline NIHSS score (ΔAUC = 0.111, *p* = 0.046). However, no significant differences were found between the baseline NIHSS score and platelet count (*p* = 0.071), nor between fibrinogen level and platelet count (*p* = 0.832) (Table 7).

ROC analysis showed (Table 8) that the biomarker model had the lowest discriminative ability (AUC = 0.682; 95% CI 0.624–0.737). The full model achieved good discrimination (AUC = 0.738; 95% CI 0.682–0.789), while the reduced model showed a comparable performance (AUC = 0.739; 95% CI 0.683–0.790), indicating limited incremental value of baseline NIHSS score. The extended model incorporating admission glucose showed a similar AUC (0.742; 95% CI 0.686–0.793), suggesting minimal improvement in predictive performance.

Pairwise DeLong comparisons (Table 9) demonstrated no significant difference between the full and reduced models (ΔAUC = 0.001, *p* = 0.804; adjusted *p* = 0.851), indicating comparable discriminative performance. Both the full and reduced models showed significantly higher AUCs than the baseline NIHSS score and the biomarker model, a significant difference was also observed between the NIHSS score and the biomarker model (*p* = 0.008). These differences remained statistically significant after Benjamini–Hochberg correction, indicating superior performance of the biomarker model over clinical severity alone.

In contrast, neither the full nor the reduced model significantly outperformed baseline ASPECTS alone (*p* = 0.120 and *p* = 0.112, respectively; adjusted *p* = 0.154 for both), suggesting that imaging-based assessment already captures substantial predictive information. Furthermore, the addition of admission glucose to the reduced model resulted in a negligible and non-significant change in discrimination (ΔAUC = 0.0026, 95% CI −0.0245 to 0.0297; *p* = 0.851), indicating no incremental predictive value.

Table 10 summarizes model performance based on AUC and DeLong pairwise comparisons. The reduced and full models were jointly ranked highest, with no statistically significant difference in discriminative ability, although the reduced model was preferred due to its greater parsimony. Baseline ASPECTS and the biomarker model demonstrated comparable discriminative performance, with no statistically significant difference between them. Baseline NIHSS score showed the lowest discriminative ability among all models. Overall, the reduced model provided the most balanced trade-off between predictive performance and model simplicity.

Table 11 presents the optimal cut-off values and diagnostic performance of individual predictors and the reduced model. Baseline ASPECTS showed high sensitivity but low specificity at the optimal threshold (≤8). Fibrinogen and platelet count demonstrated moderate and complementary performance, with fibrinogen showing higher sensitivity and platelet count higher specificity. The reduced model achieved the best overall diagnostic performance, as reflected by the highest Youden index and higher specificity.

## 4. Discussion

The blood–brain barrier (BBB) plays a central role in the pathophysiology of hemorrhagic transformation (HT) following reperfusion. Disruption of the BBB during the acute ischemic phase leads to increased vascular permeability, extravasation, and secondary hemorrhage. The incidence of HT in our study (19%) was lower than that reported in previous studies (36–49.5%) [12,29,30], which may reflect differences in patient characteristics and interventional strategies.

In this study, we identified Baseline ASPECTS, fibrinogen level, and platelet count as independent predictors of HT after MT. Combined models improved discriminative performance compared with individual variables; however, the reduced model achieved comparable performance to the full model and did not significantly outperform baseline ASPECTS alone, underscoring the central role of imaging-based assessment. Baseline ASPECTS demonstrated the highest discriminative ability among individual predictors and provided a clear and clinically applicable threshold (≤8). This finding is consistent with prior studies demonstrating that lower baseline ASPECTS reflects a larger infarct burden and is associated with worse clinical outcomes, thereby increasing the risk of HT following reperfusion [9,31,32]. Mechanistically, extensive ischemic injury is associated with endothelial dysfunction and increased matrix metalloproteinase-9 (MMP-9) activity, which compromises BBB integrity. This likely explains the strong and robust predictive performance of ASPECTS, even when compared with multivariable models [7,33]. Lower fibrinogen levels and reduced platelet counts were also independently associated with HT in our cohort [22,34]. Several studies have demonstrated that reduced plasma fibrinogen availability is associated with an increased risk of bleeding complications [35,36]. Similarly, thrombocytopenia has been associated with a higher risk of intracranial hemorrhage following reperfusion therapies [18,19]. A study by Changchun Lin et al. (2021) reported that fibrinogen < 2.165 g/L and platelet count < 171.5 ×10^9^/L predicted HT with a specificity of 77.9% and a sensitivity of 55.1% [29]. However, the clinical implication of fibrinogen reduction may depend on the baseline fibrinogen profile of each population. In our cohort, the identified fibrinogen cut-off likely represents a relative reduction in fibrinogen reserve rather than absolute hypofibrinogenemia. This relative change may reflect a coagulation profile associated with increased susceptibility to bleeding after reperfusion. In contrast to previous studies reporting lower fibrinogen thresholds associated with hemorrhagic complications, our ROC-derived cut-off (≤3.38 g/L) was higher. This difference may be explained by variations in baseline fibrinogen levels, patient characteristics, timing of measurement, and treatment conditions. Therefore, this threshold should be interpreted as a cohort-specific predictive marker rather than a universal biological threshold for hypofibrinogenemia. External validation in independent cohorts with different baseline fibrinogen distributions is required before clinical application. Therefore, our findings should be considered as a risk stratification indicator within similar patient populations rather than a universal clinical threshold.

Our ROC analysis suggests a complementary pattern among these predictors: Baseline ASPECTS demonstrated high sensitivity, platelet count showed higher specificity, and fibrinogen appeared to modulate overall risk.

Both the full and reduced models achieved similar discriminative performance (AUC ≈ 0.74). However, DeLong testing revealed no significant difference between the two models, and neither significantly outperformed baseline ASPECTS alone. This suggests that most of the predictive signal for HT is captured by imaging-based variables, while clinical and laboratory variables provide only incremental contributions. From a statistical perspective, this observation is consistent with a “ceiling effect,” whereby a highly informative variable explains a substantial proportion of outcome variance [37]. Consequently, adding further variables yields only marginal improvements in AUC that often fails to reach statistical significance, reflecting the principle of diminishing returns in multivariable modeling. Although admission glucose was associated with HT in univariate analysis, its inclusion did not improve the performance of the reduced model, indicating no meaningful incremental predictive value beyond baseline ASPECTS, fibrinogen level, and platelet count.

From a biological standpoint, the variables in our models represent two distinct mechanistic axes: structural brain injury (ASPECTS) and systemic hemostatic status (fibrinogen and platelet count). Our findings suggest that structural injury appears to be a major determinant of HT risk, whereas hematologic factors primarily modulate this risk. Thus, when BBB disruption is already substantial, the likelihood of HT remains high regardless of coagulation status, limiting the incremental value of biomarkers in overall prediction [38,39]. From a methodological perspective, the reduced model offers clear practical advantages. With comparable performance but fewer variables, it reduces the risk of overfitting, enhances model stability, and improves generalizability to external populations. The principle of parsimony supports the selection of simpler models when predictive performance is equivalent, particularly in time-sensitive clinical settings.

Although the addition of biomarkers did not significantly improve AUC, they may still provide clinically meaningful information in specific scenarios, particularly in patients with borderline ASPECTS values (e.g., 7–8), where treatment decisions may be uncertain. In such cases, fibrinogen and platelet count may help refine risk stratification and support individualized decision-making. Therefore, the lack of statistical significance in DeLong comparisons does not imply a lack of clinical utility [40].

The baseline ASPECTS threshold of ≤8 provides a simple and practical cutoff for identifying patients at increased risk of HT. In contrast, the thresholds for fibrinogen and platelet count are data-driven and may vary across populations, warranting cautious interpretation. The optimal predicted probability threshold of the reduced model reflects the balance between sensitivity and specificity but requires external validation before widespread clinical implementation [9,41].

### Limitations

This study has several limitations. First, it was conducted at a single center with a moderate sample size, which may limit generalizability. Second, external validation was not performed, and the model requires confirmation in independent cohorts. Third, the identified biomarker thresholds were data-driven and may be population-specific. Finally, additional imaging parameters (e.g., CT perfusion or blood–brain barrier permeability) and other inflammatory or endothelial biomarkers were not included, which may further enhance predictive performance in future studies. Imaging assessment was limited to ASPECTS, while other non-contrast CT thrombus-related markers (e.g., hyperdense MCA sign) were not available. Their absence may reduce the completeness of the imaging-based predictive framework. The inclusion criteria (ASPECTS ≥ 6, NIHSS ≥ 5, and successful reperfusion [mTICI ≥ 2b]) may limit the generalizability of our findings. The ASPECTS cutoff (≤8) was derived from a restricted range (6–10) and requires further validation in patients with lower ASPECTS scores.

## 5. Conclusions

In patients with acute ischemic stroke undergoing mechanical thrombectomy, baseline ASPECTS, fibrinogen level, and platelet count were independently associated with hemorrhagic transformation. Among individual predictors, baseline ASPECTS demonstrated the strongest discriminative ability, highlighting the central role of imaging-based assessment in evaluating HT risk. Although combined models incorporating hematologic biomarkers improved overall predictive performance, the reduced model (baseline ASPECTS, fibrinogen level, and platelet count) achieved comparable discrimination to the full model while maintaining greater simplicity and practicality. These findings suggest that structural ischemic injury is the dominant determinant of HT risk, whereas fibrinogen and platelet count provide complementary information reflecting systemic hemostatic status. The reduced model may therefore serve as a practical tool for risk stratification, particularly in patients with borderline ASPECTS values where treatment decisions remain uncertain. However, external validation in larger multicenter cohorts is required before routine clinical implementation.

## Figures and Tables

**Table 1:** Basic characteristics of the study groups.

Characteristics	Non-HT Group (*n* = 222)	HT Group (*n* = 52)	Total (*n* = 274)	*p*-Value	Adjusted *p*-Value (BH)
Sex (male), *n* (%)	139 (62.6)	35 (67.3)	174 (63.5)	0.527 *	0.791
Age (year), M (IQR)	69 (60–76)	70.5 (65.3–77)	69.0 (62.0–77.0)	0.133	0.508
Medical history					
Smoking, *n* (%)	7 (3.2)	1 (1.9)	8 (2.9)	1.0 **	1.000
Hypertension, *n* (%)	123 (55.4)	32 (61.5)	155 (56.6)	0.422 *	0.772
Diabetes mellitus, *n* (%)	54 (24.3)	14 (26.9)	68 (24.8)	0.696 *	0.833
Dyslipidemia, *n* (%)	65 (29.3)	23 (44.2)	88 (32.1)	0.038 *	0.228
History of stroke or TIA, *n* (%)	52 (23.4)	9 (17.3)	61 (22.3)	0.340 *	0.772
Coronary artery disease, *n* (%)	11 (5.0)	3 (5.8)	14 (5.1)	0.734 **	0.833
Atrial fibrillation, *n* (%)	50 (22.5)	16 (30.8)	66 (24.1)	0.211 *	0.681
Cancer	7 (3.2)	0 (0.0)	7 (2.6)	0.353 **	0.772
Anticoagulant agent, *n* (%)	16 (7.2)	6 (11.5)	22 (8.0)	0.392 **	0.772
Prior antiplatelet therapy	19 (8.6)	9 (17.3)	28 (10.2)	0.061 *	0.312
TOAST					
LAA, *n* (%)	161 (72.5)	36 (69.2)	197 (71.9)	0.683 **	0.833
CE, *n* (%)	60 (27.0)	16 (30.8)	76 (27.7)		
Other (arterial dissection), *n* (%)	1 (0.5)	0 (0.0)	1 (0.4)		
Baseline NIHSS score, M (IQR)	13.0 (8.0–17.0)	14.0 (10.0–17.50)	14.00 (8.0–17.0)	0.379 ^#^	0.772
10-day NIHSS score, M (IQR)	7.0 (3.0–14.0)	24.50 (9.3–32.0)	8.0 (3.8–18.5)	<0.001 ^#^	0.011
Baseline ASPECTS, M (IQR)	8.0 (7.0–10.0)	7.0 (6.0–8.0)	8.0 (7.0–9.0)	<0.001 ^#^	0.011
Discharge mRS score, M (IQR)	3.0 (2.0–5.0)	5.0 (4.0–5.0)	4.0 (2.0–5.0)	<0.001 ^#^	0.011
3-month mRS score (≤2), *n* (%)	92 (41.4)	18 (34.6)	110 (40.1)	0.366 *	0.772
Leukocyte, ×10^9^/L, M (IQR)	10.1 (8.1–13.1)	11.5 (9.2–14.8)	10.6 (8.3–13.4)	0.067 ^#^	0.312
Platelet, ×10^9^/L, M (IQR)	243.0 (208.0–296.0)	212 (186.5–264.3)	240.0 (202.0–291.3)	0.002 ^#^	0.017
Red blood cell, ×10^12^/L, M (IQR)	4.8 (4.4–5.1)	4.7 (4.4–5.1)	4.8 (4.4–5.1)	0.923 ^#^	0.994
Hemoglobin, g/L, M (IQR)	143.0 (131.0–153.0)	142.0 (133.0–151.8)	143.0 (131.0–153.0)	0.724 ^#^	0.833
PT, s, M (IQR)	11.5 (10.7–12.7)	11.9 (10.80–13.2)	11.6 (10.8–12.8)	0.231 ^#^	0.693
INR, M (IQR)	1.0 (0.9–1.1)	0.98 (0.92–1.0)	0.99 (0.9–1.1)	0.904 ^#^	0.994
PTT, s, M (IQR)	28.2 (26.2–30.8)	28.3 (26.0–30.5)	28.2 (26.1–30.6)	0.512 ^#^	0.791
Fibrinogen, g/L, M (IQR)	3.8 (3.3–4.3)	3.3 (2.9–4.0)	3.7 (3.2–4.3)	0.001 ^#^	0.011
Glucose, mmol/L, M (IQR)	7.2 (6.1–9.1)	6.8 (5.8–7.8)	7.1 (5.9–8.7)	0.033 ^#^	0.228
Creatinine, µmol/L, M (IQR)	74.0 (62.0–90.2)	76.0 (61.2–96.0)	74.0 (62.0–92.0)	0.426 ^#^	0.772
Urea, mmol/L, M (IQR)	5.9 (4.9–7.9)	6.2 (5.3–7.8)	6.0 (5.0–7.9)	0.454 ^#^	0.772
ALT, U/L, M (IQR)	19.0 (13.7–28.4)	20.9 (12.3–30.0)	19.6 (13.0–28.8)	0.995 ^#^	1.000
AST, U/L, M (IQR)	27.0 (22.0–34.7)	25.7 (21.0–34.6)	26.8 (21.7–34.7)	0.555 ^#^	0.804
Sodium, mmol/L, M (IQR)	137.0 (135.2–139.0)	136.7 (135.2–139.6)	137.0 (135.2–139.0)	0.711 ^#^	0.833
Potassium, mmol/L, M (IQR)	3.7 (3.5–3.9)	3.7 (3.5–4.0)	3.7 (3.5–3.9)	0.981 ^#^	1.000
Cholesterol, mmol/L, M (IQR)	5.0 (4.44–5.9)	5.0 (4.6–6.0)	5.0 (4.5–5.9)	0.660 ^#^	0.833
Triglyceride, mmol/L, M(IQR)	1.7 (1.1–2.5)	1.5 (1.1–3.0)	1.6 (1.1–2.6)	0.670 ^#^	0.833
HDL_C, mmol/L, M (IQR)	1.1 (0.9–1.3)	1.1 (1.0–1.3)	1.1 (1.0–1.3)	0.688 ^#^	0.833
LDL_C, mmol/L, M (IQR)	3.1 (2.6–3.7)	3.1 (2.7–3.7)	3.1 (2.6–3.7)	0.436 ^#^	0.772
T1, h, M (IQR)	267.5 (191.7–426.2)	327.5 (266.2–417.2)	287.0 (196.7–425.0)	0.114 ^#^	0.479
T2, h, M (IQR)	33.0 (21.7–48.0)	40.0 (25.0–48.0)	35.0 (22.7–48.0)	0.352 ^#^	0.772
T3, h, M (IQR)	99.0 (80.0–133.5)	100.0 (85.0–127.7)	99.5 (80.0–133.0)	0.468 ^#^	0.772
T4, h, M (IQR)	25.0 (15.0–35.0)	29.5 (20.0–35.0)	25.0 (15.0–35.0)	0.478 ^#^	0.772
T5, h, M (IQR)	441.5 (320.0–607.0)	448.0 (357.5–583.2)	445.0 (320.7–605.0)	0.196 ^#^	0.681

HT, hemorrhagic transformation; M, median; IQR, interquartile range; S, second; TIA, transient ischemic attack; TOAST, Trial of Org 10172 in Acute Stroke Treatment; LAA, large-artery atherosclerosis; CE, cardioembolism; NIHSS, National Institutes of Health Stroke Scale; ASPECTS, Alberta Stroke Program Early CT Score; PT, prothrombin time; INR, international normalized ratio; ALT, Alanine Aminotransferase; AST, Aspartate Aminotransferase; T1, time from symptom onset to hospital arrival; T2, time from hospital arrival to confirmed diagnosis; T3, time from hospital arrival to puncture; T4, time from puncture to vascular recanalization; T5, time from symptom onset to vascular recanalization; mRS, modified Rankin Scale; BH, Benjamini–Hochberg. * *p*-value from chi-square test; ** *p*-value from Fisher’s exact test; # *p*-value from Mann–Whitney U test.

**Table 2:** Comparison of baseline characteristics among HT subgroups.

Characteristics	HI (*n* = 21)	PH (*n* = 31)	*p*-Value	Adjusted *p*-Value (BH)
Sex (male), *n* (%)	13 (61.9)	22 (71.0)	0.494 *	0.719
Age (year), M (IQR)	69.0 (64.5–77.0)	73.0 (66.0–78.0)	0.472 ^#^	0.719
Dyslipidemia, *n* (%)	10 (47.6)	13 (41.9)	0.686 *	0.784
Atrial fibrillation, *n* (%)	6 (28.6)	10 (32.3)	0.777 *	0.816
Anticoagulant agent, *n* (%)	3 (14.3)	3 (9.7)	0.675 **	0.784
Baseline NIHSS score, M (IQR)	12.0 (9.0–15.0)	14.0 (12.0–18.0)	0.086 ^#^	0.291
10-day NIHSS score, M (IQR)	12.0 (6.0–32.0)	32.0 (15.0–40.0)	0.007 ^#^	0.112
Baseline ASPECTS, M (IQR)	8.0 (6.50–8.0)	7.0 (6.0–8.0)	0.359 ^#^	0.719
Discharge mRS score, M (IQR)	4.0 (3.50–5.0)	5.0 (4.0–6.0)	0.017 ^#^	0.136
3-month mRS score (mRS ≤ 2), *n* (%)	6 (28.6)	12 (38.7)	0.451 *	0.719
Platelet, ×10^9^/L, M (IQR)	192.0 (177.0–270.50)	216.0 (189.0–251.0)	0.576 ^#^	0.768
Fibrinogen, g/L, M (IQR)	3.4 (2.9–4.2)	3.3 (2.9–3.9)	0.371 ^#^	0.719
Glucose, mmol/L, M (IQR)	6.9 (5.8–8.2)	6.5 (5.4–7.5)	0.401 ^#^	0.719
T5, h, M (IQR)	485.0 (355.0–626.5)	435.0 (355.0–585.0)	0.816 ^#^	0.816
sICH, *n* (%)	3 (14.3)	11 (35.5)	0.091 *	0.291
In-hospital mortality	1 (4.8)	8 (25.8)	0.067 **	0.291

HI, hemorrhagic infarction; PH, parenchymal hematoma; M, median; IQR, interquartile range; NIHSS, National Institutes of Health Stroke Scale; ASPECTS, Alberta Stroke Program Early CT Score; T5, time from symptom onset to vascular recanalization; mRS, modified Rankin Scale; BH, Benjamini–Hochberg; sICH, symptomatic intracranial hemorrhage. * *p*-value from chi-square test; ** *p*-value from Fisher’s exact test; # *p*-value from Mann–Whitney U test.

**Table 3:** Univariate and multivariate logistic regression analyses of risk factors for HT.

Parameters	Univariate		Multivariate			
	OR (95% CI)	*p*-Value	Adjusted OR (95% CI)	β Coefficient (B)	SE	*p*-Value
Intercept (Constant)				4.920	1.209	<0.001
Baseline ASPECTS (score)	0.75 (0.63–0.88)	0.001	0.73 (0.61–0.88)	−0.310	0.093	0.001
Platelet count (×10^9^/L)	0.992 (0.987–0.997)	0.002	0.993 (0.988–0.999)	−0.007	0.003	0.018
Fibrinogen level (g/L)	0.45 (0.30–0.68)	<0.001	0.51 (0.32–0.79)	−0.683	0.226	0.003

ASPECTS, Alberta Stroke Program Early CT Score.

**Table 4:** Bootstrap validation of multivariable logistic regression model.

Variable	B	Bootstrap 95% CI	*p*-Value
Fibrinogen level (g/L)	−0.683	−1.119 to −0.294	0.002
Platelet count (×10^9^/L)	−0.007	−0.012 to −0.002	0.016
Baseline ASPECTS	−0.310	−0.505 to −0.167	0.001

**Table 5:** Bootstrap validation of the final model.

Variable	β	Bias	Bootstrap SE	*p*-Value	BCa 95% CI
Baseline ASPECT	−0.310	−0.008	0.091	0.001	−0.514 to −0.166
Fibrinogen level (g/L)	−0.683	−0.021	0.235	0.001	−1.103 to −0.281
Platelet count (×10^9^/L)	−0.007	0.000	0.003	0.012	−0.012 to −0.002

**Table 6:** AUC of each marker.

Marker	AUC	95% CI	SE
Baseline ASPECTS	0.684	0.625–0.738	0.036
Baseline NIHSS score	0.539	0.478–0.599	0.040
Fibrinogen level (g/L)	0.650	0.590–0.706	0.043
Platelet count (×10^9^/L)	0.639	0.579–0.696	0.043

**Table 7:** Pairwise DeLong comparison of individual predictors.

Comparison	ΔAUC	Z	*p*-Value	95% CI
Baseline ASPECTS vs. baseline NIHSS score	0.145	3.164	0.002	0.055 to 0.234
Baseline ASPECTS vs. fibrinogen level	0.034	0.645	0.519	−0.070 to 0.138
Baseline ASPECTS vs. platelet count	0.045	0.830	0.407	−0.061 to 0.151
Baseline NIHSS score vs. fibrinogen level	0.111	1.998	0.046	0.002 to 0.219
Baseline NIHSS score vs. platelet count	0.100	1.805	0.071	−0.009 to 0.208
Fibrinogen level vs. platelet count	0.011	0.212	0.832	−0.089 to 0.112

Note: vs.: versus.

**Table 8:** ROC curve analysis of models for HT after MT (*n* = 274).

Model	AUC	95% CI	SE
Biomarker model	0.682	0.624–0.737	0.042
Full model	0.738	0.682–0.789	0.039
Reduced model	0.739	0.683–0.790	0.039
Extended model	0.742	0.686–0.793	0.040

Note: Full model, baseline ASPECTS + baseline NIHSS score + fibrinogen level + platelet count; reduced model, baseline ASPECTS + fibrinogen level + platelet count; biomarker model, fibrinogen level + platelet count; extended model, reduced model + admission glucose.

**Table 9:** Pairwise DeLong comparison of predictive models.

Comparison	ΔAUC	Z Value	95% CI	*p*-Value	Adjusted *p*-Value (BH)
Full model vs. Reduced model	0.001	0.248	−0.01 to 0.012	0.804	0.851
Full model vs. baseline NIHSS score	0.199	3.808	0.1 to 0.301	0.0001	<0.001
Full model vs. Biomarker model	0.056	2.359	0.009 to 0.102	0.018	0.032
Reduced model vs. baseline NIHSS score	0.20	4.099	0.104 to 0.296	<0.0001	<0.001
Reduced model vs. Biomarker model	0.057	2.513	0.013 to 0.124	0.012	0.027
Baseline NIHSS score vs. Biomarker model	0.143	2.648	0.0372 to 0.249	0.008	0.024
Full model vs. baseline ASPECTS	0.0542	1.557	−0.014 to 0.123	0.120	0.154
Reduced model vs. baseline ASPECTS	0.0556	1.589	−0.013 to 0.124	0.112	0.154
Reduced model vs. extended model	0.0026	0.188	−0.0245 to 0.0297	0.851	0.851

Note: Full model, baseline ASPECTS + baseline NIHSS score + fibrinogen level + platelet count; reduced model, baseline ASPECTS + fibrinogen level + platelet count; biomarker model, fibrinogen level + platelet count; extended model, reduced model + admission glucose.

**Table 10:** Summary of model performance and ranking based on AUC and DeLong test comparisons.

Rank	Model	Key Finding
1	Reduced model	Best parsimonious model; no significant difference vs. full model
1	Full model	No incremental predictive value compared with reduced model
2	Baseline ASPECTS	Moderate discrimination; comparable to biomarker model
2	Biomarker model	Moderate discrimination; comparable to baseline ASPECTS
3	Baseline NIHSS score	Lowest discriminative ability

Note: Full model, baseline ASPECTS + baseline NIHSS score + fibrinogen level + platelet count; reduced model, baseline ASPECTS + fibrinogen level + platelet count; biomarker model, fibrinogen level + platelet count.

**Table 11:** Optimal cut-off values and diagnostic performance of predictors for HT.

Variable	Cut-Off	Sensitivity (%)	Specificity (%)	Youden Index
Baseline ASPECTS	≤8	84.62	41.89	0.265
Fibrinogen level	≤3.38	57.69	68.92	0.266
Platelet count	≤199	44.23	81.08	0.253
Reduced model	0.324	46.15	91.89	0.3805

Note: reduced model, baseline ASPECTS score + fibrinogen level + platelet count.

## Data Availability

The data presented in this study are available on request from the corresponding author due to appropriate ethical and confidentiality considerations.

## Abbreviations

The following abbreviations are used in this manuscript:

HT	Hemorrhagic transformation
MT	Mechanical thrombectomy
AIS	Acute ischemic stroke
ASPECTS	Alberta Stroke Program Early CT Score
sICH	Symptomatic intracranial hemorrhage
CTA	Computed tomography angiography
mTICI	Modified Thrombolysis in Cerebral Infarction
HI	Hemorrhagic infarction
NIHSS	National Institutes of Health Stroke Scale
mRS	Modified Rankin Scale
ROC	Receiver operating characteristic
AUC	Area under the curve
BBB	Blood–brain barrier
